# Clathrin-Mediated Endocytosis and Adaptor Proteins

**Published:** 2013

**Authors:** N.V. Popova, I.E. Deyev, A.G. Petrenko

**Affiliations:** Shemyakin-Ovchinnikov Institute of Bioorganic Chemistry of the Russian Academy of Sciences, Miklukho-Maklaya St., 16/10, Moscow, Russia, 117997

**Keywords:** adaptor proteins, clathrin, endocytosis

## Abstract

Macromolecules gain access to the cytoplasm of eukaryotic cells using one of several ways of which clathrin-dependent endocytosis is the most researched. Although the mechanism of clathrin-mediated endocytosis is well understood in general, novel adaptor proteins that play various roles in ensuring specific regulation of the mentioned process are being discovered all the time. This review provides a detailed account of the mechanism of clathrin-mediated internalization of activated G protein-coupled receptors, as well as a description of the major proteins involved in this process.

## INTRODUCTION

Endocytosis is a fundamental process that ensures delivery of extracellular or membrane-localized macromolecules to the cytoplasm. Endocytosis is necessary for nutrients to reach the cell, the regulation of the activity of transmembrane receptors, and synaptic vesicle recycling. Clathrin-mediated endocytosis represents the entry of fragments of the cytoplasmic membrane, along with all of their contents, into the cell in the form of vesicles coated on the outside with a lattice consisting of polymerized clathrin. In particular, the clathrin-mediated mechanism is utilized to carry out the endocytosis of activated cell surface receptors. Binding of the receptor molecule to the ligand and activation of the former render possible the subsequent binding of the intracellular part of the receptor to the adaptor proteins. These proteins mediate the interaction between receptors and clathrin molecules, resulting in the formation of the clathrin coat. Several classes of adaptor proteins have been identified.

## ENDOCYTOSIS OF G-PROTEIN-COUPLED RECEPTORS AS AN EXAMPLE OF CLATHRIN-MEDIATED ENDOCYTOSIS

The superfamily of G-protein-coupled receptors (GPCR ) is considered to be the largest family of membrane proteins involved in intracellular signal transduction [1]. The general structural feature of GPCR is the presence of seven α-helical transmembrane hydrophobic segments each consisting of 25–35 amino acid residues [2]. The N-terminal portion of GPCR and three loops between the transmembrane segments are found outside the cell, and the C-terminal part and the other three loops are found on the cytoplasmic side of the plasma membrane.

The ligands of various GPCR include ions, organic odorants, amines, peptides, proteins, lipids, nucleotides, and photons. Activation of the receptors by their corresponding ligands leads to the formation of complexes consisting of receptors and heterotrimeric G-proteins (consisting of 3 subunits) and the associated exchange of GDP for GTP. This exchange causes the dissociation of a G-protein into a GTP-bound α-subunit and a complex consisting of β- and γ-subunits, as well as the dissociation of all three subunits of the G-protein from the receptor. It is now proven that both the α-subunit and the βγ complex serve as signal transducers by activating or inhibiting enzymes and ion channels [3]. Bound GTP is hydrolyzed following interaction with the effector and re-association of the α-subunit and βγ to form a complex consisting of three subunits with a bound GDP. This complex is again able to interact with the activated receptor [4].

Binding of the ligand to the receptor leads to conformational changes that give rise to the G-protein-mediated signal transduction and conversion of the receptor into the protein kinase GRK substrate (G-protein-coupled Receptor Kinases). The serine or threonine residues of the ligand-activated receptor located in the cytoplasmic domain and/or in the third cytoplasmic loop are phosphorylated. Then, β-arrestins bind to the activated and phosphorylated receptor [5]. β-Arrestins play a significant role in the process of internalization of GPCR as their binding leads to clathrin-mediated endocytosis of the receptor attributed to interaction with the components of the endocytotic mechanism – clathrin and the AP-2 adaptor protein complex [6, 7].

**Fig. 1:**
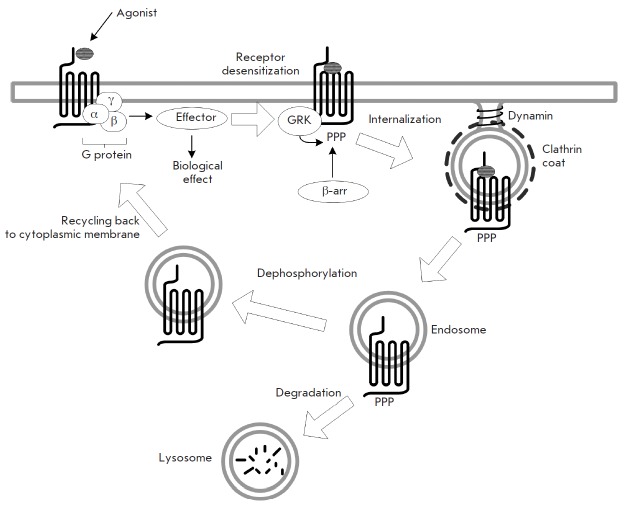
Schematic diagram of clathrin-mediated internalization of a receptor following its activation

Newly formed clathrin-coated vesicles containing the receptor detach from the cytoplasmic membrane by means of the protein called dynamin tightening the neck of the forming vesicles [8]. The internalized receptor- ligand complex detached as part of the vesicles further undergoes intracellular transport. The first stage of this pathway is the formation of early endosomes. It is believed that canonical early endosomes contain a small GTPase Rab5 and the early endosome antigen 1 (EE A1). In the majority of cases, the internalized receptor remains accessible to the molecules of the intracellular signaling cascade and, therefore, can continue to participate in the signal transduction as if it was localized on the surface of the membrane [9]. Then, depending on the type of the receptor, one of two scenarios is possible. The receptor either dissociates from the bound ligand and is recycled back to the cell surface (re-sensitization) or is transferred to late endosomes and is subjected to degradation within lysosomes (*Fig. 1*). The fate of specific receptors depends on whether the activation was of a short-term nature or was a prolonged type of activation/reactivation [10]. Thus, for instance, the β2- adrenergic receptor following short-term activation by an agonist recycles back primarily to the cytoplasmic membrane; however, upon prolonged activation it can be transferred to the lysosome for degradation, thereby reducing the number of receptors on the surface of the membrane (down-regulation) [10].

Recycling to the cytoplasmic membrane can occur via a rapid pathway through Rab4-containing endosomes, and via a slow pathway through Rab11-containing recycling endosomes [11]. It is believed that late endosomes contain receptors intended for degradation. Transition from early to late endosomes is accompanied by the replacement of protein Rab5 with protein Rab7 – the so-called “Rab conversion” [12].

## MECHANISM OF CLATHRIN-MEDIATED INTERNALIZATION

Clathrin-coated vesicles have a three-layered structure: the outer layer is formed by clathrin (clathrin lattice), the internal layer is a lipid membrane with protein inclusions, while adaptor proteins are found in between. The adaptor protein complexes interact directly with the lipid bilayer, and clathrin in turn binds to the adaptors [13].

It is presumed that endocytosis begins with the formation of pits on the inner surface of the cytoplasmic membrane containing clathrin, the AP-2 adaptor protein complex, and accessory proteins [14]. The subunits of the adaptor complex trigger the formation of the clathrin lattice at specific sites of the cytoplasmic membrane and mediate the interaction between clathrin and the cargo protein [15]. AP-2 plays an important role in selecting the target for endocytosis by binding either directly to a transmembrane cargo protein containing the necessary sequences or via helper proteins, such as β-arrestins [16]. Binding of AP-2 to the membrane is a two-stage process. First the α-subunit of AP-2 binds weakly to phosphatidylinositol-4,5-bisphosphate (PtdIns(4,5)P2). AP-2 affinity for the corresponding endocytic motifs increases upon phosphorylation of a threonine residue in the μ2-subunit of AP-2 [17] by adaptor-associated kinase, AAK1 [18, 19]. This phosphorylation enables the μ2-subunit to bind to motifs of the cargo protein undergoing endocytosis and to PtdIns(4,5)P2 in the membrane creating the foundation for the formation of a clathrin-coated vesicle. Then, the adaptor complex can bind to the other accessory proteins, such as CALM, required for the formation of a clathrin lattice. Removal of this protein from the cell leads to the formation of large asymmetric clathrincoated pits [20]. Simultaneously with the polymerization of clathrin, the process involves a variety of other proteins required for the control of the invagination of the cytoplasmic membrane and the formation of the pit on it. It is believed that the bending of the membrane is attributed to the action of several proteins containing BAR-domains (Bin/amphiphysin/Rvs) [21], such as amphiphysin [22] and endophilin [23]. The protein epsin is also able to stimulate the bending of the membrane [24]. Polymerizing clathrin forms the lattice (consisting of hexagons and pentagons) surrounding the emerging pits and, thus, stabilizes the membrane curvature [25].

Subsequent deformation of the membrane and polymerization of clathrin lead to the clathrin-coated vesicle remaining attached to the main part of the membrane via a narrow neck requiring GTPase dynamin for the completion of the detachment of the vesicle. Amphiphysin already being a part of the vesicle contains binding sites for both clathrin and dynamin. It is presumed that it “attracts” dynamin to the forming vesicle and facilitates its oligomerization [26]. According to the two proposed models, after polymerization of dynamin around the neck of the vesicle, a GTP hydrolysis-dependent change in its structure results in the constriction (first model) or stretching (second model) of the neck and detachment of the vesicle from the remainder of the membrane [27].

Removal of the clathrin coat from the surface of the vesicle is necessary for further fusion of the vesicle with the target membrane and delivery of endocytosed “cargo” to the target destination. The primary participants in the process of depolymerization of the clathrin coat include the proteins Hsc70 and auxilin. Auxilin, an homologue of Hsp40, binds to clathrin and attracts Hsc70, which interacts with its J-domain. As a result of interaction with auxilin, the ATPase activity of Hsc70 increases and it binds to clathrin with increased affinity, thus distorting the conformation and contributing to the dismantling of the clathrin lattice into individual molecules [28, 29]. The layer formed by the adaptor complex is removed as a result of dephosphorylation of the AP-proteins by phosphatases, as it was shown for the μ1-subunit of the AP-1 [30]. The proteins synaptojanin and endophilin dephosphorylate membrane phospholipids, thereby reducing the affinity of the adaptors for vesicles [31].

## KEY PROTEINS INVOLVED IN CLATHRIN-MEDIATED ENDOCYTOSIS

**G-protein-coupled receptor kinases and β-arrestins**

A large number of proteins that are capable of direct interactions with the GPCR have been described [32]. However, only two classes of proteins, other than G-proteins, that specifically interact with ligand-activated receptors are known: GPCR kinases (GRK) and β-arrestins [33].

The GRK family comprises products of seven different genes. The expression of GRK1 and -7 is limited to retinal rods and cones, respectively. GRK4 is exclusively expressed in the cerebellum, testis and kidneys. GRK2, -3, -5 and -6, in contrast, are expressed in vari ous mammalian tissues. Seven kinases are divided into three subfamilies with respect to amino acid sequence homology. GRK1 and -7; GRK2 and -3 contain pleckstrin homology (PH) domain, and association of these kinases with plasma membranes is dependent on the interaction with the G_βγ_-subunit of the G-proteins and PtdIns(4,5)P2; GRK4–6 proteins are continuously associated with the membrane [34].

Arrestins comprise 4 proteins. Arrestins 1 and 4 (x-arrestin) are expressed in retinal rods and cones, respectively. Arrestins 2 and 3 (also known as β-arrestins 1 and 2) are present in all tissues [5]. GRK and arrestins control GPCR activity at three levels: (1) silencing – functional detachment of the receptor from its G-protein; (2) regulation of transport – removal of the receptor from the cytoplasmic membrane (internalization), recycling back to the membrane and/or degradation; and (3) signal transduction – activation or inhibition of the intracellular signaling pathways independent of G-proteins. The N-terminal portion of arrestin 1 [35] and arrestins 2 and 3 [36, 37] contains the regions responsible for the recognition of agonist-activated phosphorylated GPCR . According to the proposed model, the charged phosphate groups of the receptor destroy the polar core of the arrestin resulting in the release of its C-terminal part, which is responsible for binding to the proteins involved in endocytosis – clathrin and AP-2 [38].

**Clathrin**

**Fig. 2:**
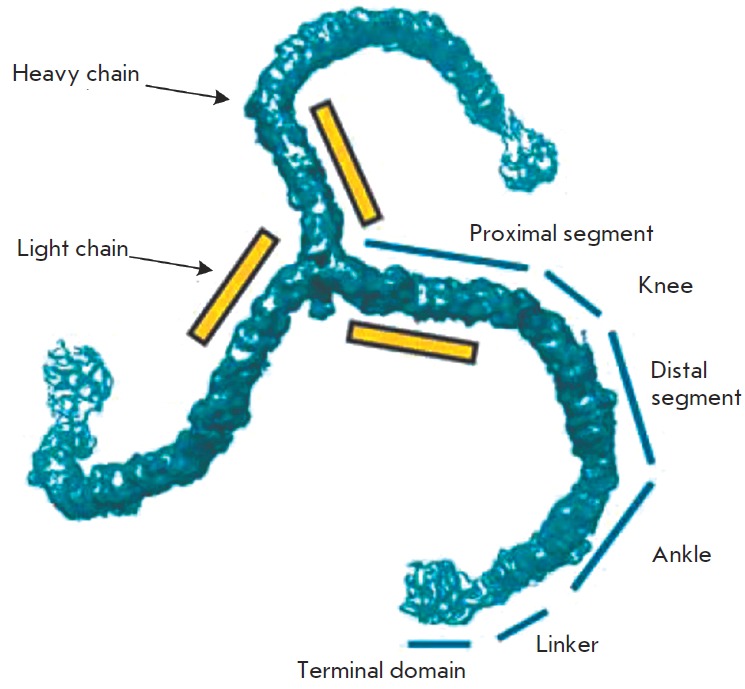
Clathrin molecule (triskelion). Segments of the clathrin heavy chain are indicated. The terminal domain is the N-terminal domain and the C-terminal domains are localized in the center of the molecule. The position of the light chains is shown schematically. Figure adapted from [40]

The major protein of clathrin-coated vesicles isolated by Pierce [39] was named clathrin as it was characterized by an ability to form structures with an ordered lattice, or “clathrates.” The clathrin molecule resembles a triskelion (derived from Greek. τρισκελης– three-legged – a symbolic mark resembling three running legs that extend from one point) and consists of three heavy and three light chains [40] (*Fig. 2*).

A clathrin heavy chain (HC) isolated from a rat’s brain is composed of 1,675 amino acid residues and has a molecular weight slightly exceeding 191 kDa (approximately 180 kDa as determined by denaturing polyacrylamide gel electrophoresis (SDS-PAGE)). The amino acid sequences of the clathrin heavy chain isolated from the brain of a human, rat or bovine are highly conserved (~ 99%) [41]. Clathrin heavy chains have also been isolated from the clathrin-coated vesicles of yeast [42] and plants [43].

Each clathrin heavy chain is in a complex with one of the light chains, LC_a_ or LC_b_, encoded by different genes. The amino acid sequences of the light chains are highly conserved across different species (95-98%). The electrophoretic mobility of light chains consisting of 230–250 amino acid residues in the SDS-PAGE corresponds to a molecular weight of approximately 30–40 kDa. Three domains are identified in the light chain: the conserved C-terminal, the central α-helical, and the acidic N-terminal. The homology of both chains at the amino acid sequence level reaches 60% [41]. The region consisting of 22 amino acid residues located at the N-terminus, the clathrin heavy chain binding site, the cysteine residues near the C-terminal portion, and the serine residues enriched casein kinase II phosphorylation site of the light chain LC_b_ are highly conserved [44]. Light chains bind the proximal domains of the clathrin heavy chains [45]; primary binding is provided by the amino acid residues 1267–1522 of the heavy chain, the residues 93–160 of the light chain LC_a_, and the residues 90–157 of the LC_b_ [46].

The regions that are necessary for the trimerization of heavy chains, binding of light chains, and formation of the clathrin lattice are located at the intersection of clathrin heavy chains [47, 48]. The domain that ensures the trimerization of heavy chains is localized between the amino acid residues 1488 and 1587.

Two sites for the binding of clathrin to adaptor proteins are located in the N-terminal domain. The first site interacts with peptides containing the “clathrin box” (LOXO[D/E], where O is a large hydrophobic amino acid). Examples of proteins containing such a motif include β-adaptins (the LLNLD sequence is found in β-adaptins 1 and 2, the LLDLD sequence is found in β-adaptin 3), β-arrestins 1 (LIELD) and 2 (LIEFE) and amphiphysins 1 and 2 (LLDLD) [49]. The second site binds proteins containing the W-box motif (PWXXW, where X is any amino acid); e.g., the molecules of the aforementioned amphiphysins 1 and 2 [50].

**Fig. 3:**
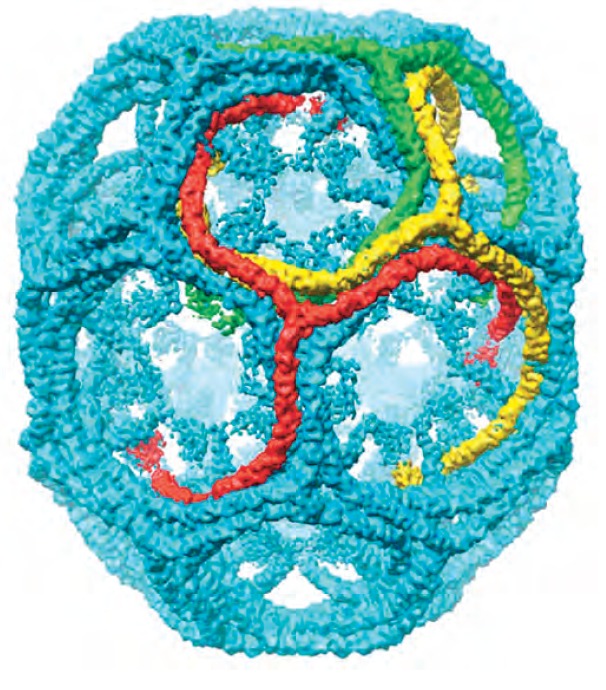
Hexagonal clathrin barrel model (7.9Å resolution). Only the heavy chains of clathrin are indicated. Figure adapted from [40]

Clathrin molecules spontaneously self-assemble in weakly acidic Ca^2+^-containing buffers with a low ionic strength to form a heterogeneous population of closed polyhedral structures resembling a lattice [51, 52]. The vertex of each triskelion is located at the vertex of the lattice. The heavy chain legs and associated light chains extend outwardly from the vertices forming the edges of the lattice (*Fig. 3*).

All heavy chains form the two adjacent edges of a polyhedral lattice. The legs appear to interact via their proximal and distal domains. Each edge consists of two antiparallel proximal domains located above two antiparallel distal domains [53]. The fragments of clathrin obtained by expression in a heterologous system and consisting of a proximal domain and a region necessary for trimerization are able to self-assemble into trimers but cannot form lattices. Formation of the clathrin lattice requires distal domains that are correctly oriented by binding of the terminal domains to adaptor proteins [54]. Terminal domains in the lattice are directed inwardly towards the center and are located under the vertex, which is positioned at a distance of two vertices from the center of the triskelion. Here, the terminal domains assume the shape of hooks-projections providing a points of contact with the inner layer formed by adaptor proteins [55].

It is worth mentioning that clathrin is also involved in mitosis. It is presumed that it is necessary for the stabilization of the microtubules that attach to kinetochores (called K-fibers) [56].

**AP adaptor protein complexes**

The second major protein of clathrin-coated vesicles is the adaptor protein complex. Its discovery was made possible by its ability to stimulate the assembly of the clathrin lattice under physiological conditions [57]. At least two adaptor complexes – AP-1 and AP-2 – have been extensively researched. These complexes have structural similarities and are composed of two different subunits of high molecular weight ~ 100 kDa (typically called adaptins), two subunits of medium size (47–50 kDa), and two low-molecular-weight subunits (17–19 kDa). The AP-2 complex comprises the following subunits: α and β2 (or β) adaptins, the μ2 subunit (50 kDa) or AP50 and the σ2 subunit (17 kDa) or AP17. The AP-1 complex comprises γ and β1 (or β’) adaptins, AP47 (or μ1) and AP19 (or σ1) [58].

The designation that uses the same letters of the Greek alphabet reflects the structural and presumably functional similarity of the subunits of the AP-1 and AP-2 complexes [59, 60]. α- and γ-adaptins differ most significantly (~ 30% amino acid sequence homology), while the μ- and σ-subunits of the AP-1 complex exhibit high levels (~ 50%) of homology to the corresponding μ- and σ-subunits of the AP-2 complex, and β1- and β2-adaptins are highly homologous (> 90%). AP-3 and AP-4 complexes that are similar in subunit composition have also been identified: δ and β3, μ3, σ3 – in the AP-3 complex; ε and β4, μ4, σ4 – in the AP-4 complex [61, 62].

**Fig. 4:**
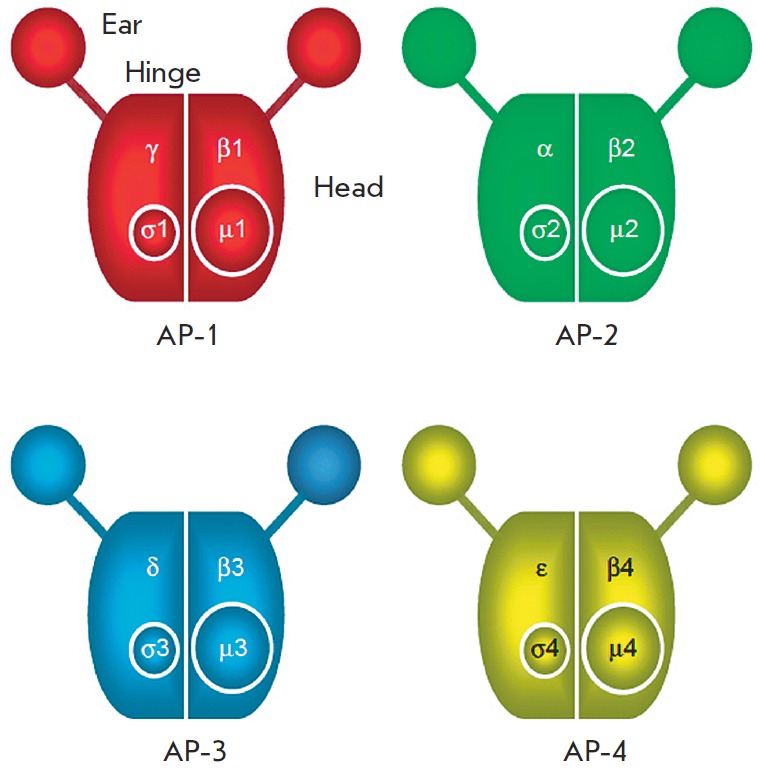
Schematic diagram of the AP complexes. All complexes consist of two large subunits, one medium subunit, and a small subunit. Figure adapted from [63]

The complex of subunits forms a structure resembling Mickey Mouse’s head (*Fig. 4*), where the center is formed by the μ and δ subunits, and the two “ears” are composed of the C-terminal domains of the two large subunits, α and β, connected to the “head” via a flexible neck [63].

While the assembly of the clathrin lattice occurs on the membrane, it has been established that clathrin itself has no affinity with lipids. It is therefore considered that clathrin is attracted to the membrane by adaptor proteins [64, 65].

AP-2 is the main protein adaptor found on the plasma membrane and is involved in the formation of clathrin-coated vesicles during endocytosis. Immunofluorescence and immunoelectron microscopy demonstrated that AP-1, -3 and -4 are localized in endosomes and the Golgi complex [66]. AP-1 mediates the transport of proteins from the Golgi complex to early or late endosomes.

AP-1 and AP-2 directly interact with the N-terminal domain of the clathrin heavy chain via the clathrinbinding site in the β-chain. In 1998, cryoelectron microscopy was utilized to elucidate the structure of the AP-2 complex with clathrin [55]. It was established that AP-2 forms a shell of continuous density in the center of the lattice. Based on the images obtained, it was also concluded that AP-2 forms contacts with the terminal domains of the clathrin lattice. Subsequently, an X-ray diffraction analysis confirmed that the β-subunits of the AP-1, -2, and -3 containing the clathrin-binding motif interact with the terminal domain of the clathrin heavy chain [49].

In addition to clathrin, AP complexes interact with integral membrane proteins. The YXXO sequence, located in the intracellular domains of many receptors, is recognized by the μ-subunit of all AP-complexes [67]. [DE]XXXL[LI] motifs, which are also found in the cytoplasmic domain of the receptors, bind to the β-subunits of the AP-complexes, and these subunits exhibit different affinities to different [DE]XXXL[LI] motifs. For instance, AP-1 and AP-2, but not AP-3, interact with the DDQRDLI and NE QLPML sites [68]. The DER APLI and EE KQPLL signals interact with AP-3, but not AP-1 or AP-2 [69].

Adaptor protein complexes are capable of binding to cell membrane lipids. Two lipid binding sites have been described [70]. The first site is located in the N-terminal part of the α-subunit of AP-2, and the second site is localized on the surface of the μ2 subunit [71]. Binding to the membrane is determined by the interaction of the phosphates PtdIns(4,5)P2 and side chains of the basic amino acid residues of the adaptor protein.

**Auxilin**

**Fig. 5:**
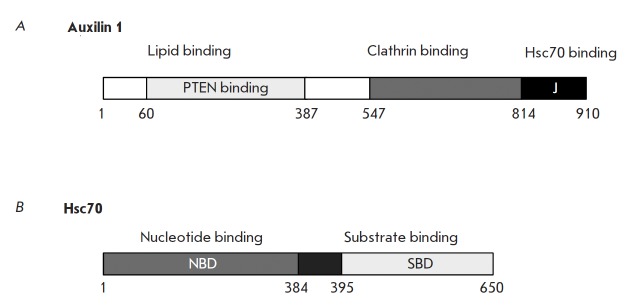
Domain organization of auxilin and Hsc70. Numbers indicate the boundaries of various domains. Figure adapted from [85]

Auxilin is a multi-domain protein with a molecular weight of 100 kDa containing a clathrin-binding domain, a J-domain, and a region homologous to phosphoinositide phosphatase PTEN (*Fig. 5A*) [72, 73]. The N-terminal domain binds to phosphoinositol derivatives and PtdIns(4,5)P2 [74, 75]. The auxilin central domain interacts with clathrin, the AP-2 complex [76], and dynamin [77].

Cryoelectron microscopy at a 20A resolution was utilized to obtain images of full-sized auxilin [78] and its fragment (549-910) [79] with a clathrin lattice. Auxilin forms a shell of density within the lattice with points of contact with the clathrin terminal domains. Auxilin is capable of interaction with the terminal domain of the clathrin heavy chain via the LLGLE motif comprising the amino acid residues 496-500. It was established that the fragment containing the J- and clathrin-binding domains of auxilin interacts with the two “ankles” of the clathrin heavy chain at their point of intersection and with the subsequent terminal domain. Binding of auxilin to the clathrin lattice causes the terminal domains to twist outwards, which is attributed to a change in the position of the “ankle.” This change in the position of terminal domains causes global changes throughout the entire lattice, increasing its diameter. It is believed that auxilin attracts Hsc70 to these areas, which are important for interaction within the lattice [55, 79].

**Hsc70**

Hsc70 is a constitutively expressed chaperone involved in many cellular processes, including protein folding, degradation, and translocation. Another interesting function of Hsc70 is its ability to “dismount” the clathrin lattice. Thus, addition of ATP and Hsc70 to clathrincoated vesicles *in vitro *causes disassembly of the clathrin lattice [80]. This is a stoichiometric reaction that requires 3 mol of Hsc70 and ATP for the dissociation of 1 mol of clathrin triskelions [80-82].

Similary to all Hsp70 family members, Hsc70 requires a protein containing a J-domain to “work” with a particular substrate [83]. Auxilin, which binds to clathrin and contains a J-domain, plays that function. The J-domain in the auxilin molecule is positioned in a way that the motif required for the interaction with the Hsc70 protein is exposed on the outside of the lattice. The QLMLT sequence (1638-1642) located in the C-terminal portion of the clathrin heavy chain is also required [84] (*Fig. 5B*). The following model of lattice disassembly is proposed: bending at the location of intersection of “ankles” caused by the interaction with auxilin enables Hsc70 to bind to its site near the C-terminus of the clathrin molecule. It is assumed that one vertex of the triskelion binds one molecule of Hsc70 and for the strong interaction to be achieved hydrolysis of ATP is required. Therefore, deformation of the clathrin lattice which had begun after binding to auxilin is enhanced [85].

**Other clathrin-interacting proteins**

**Fig. 6:**
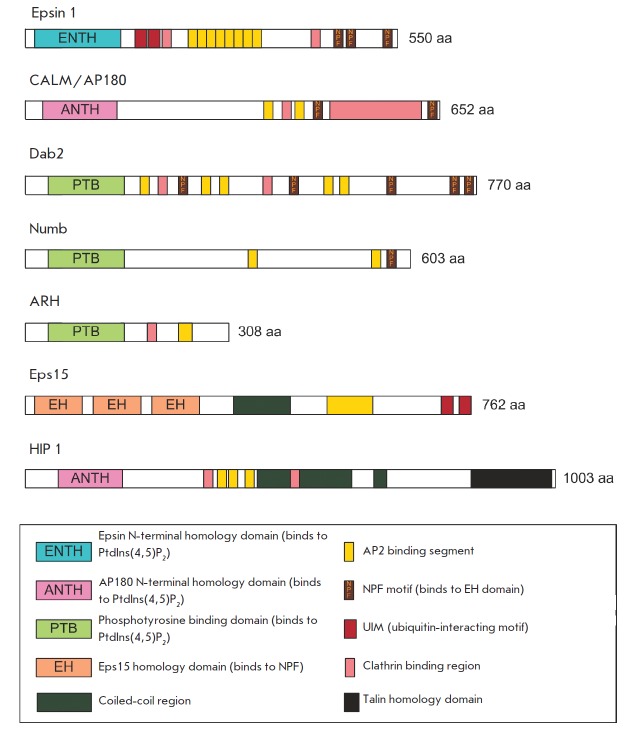
Monomeric clathrin-binding adaptors. Schematic representation of the overall domain structure. Figure adapted from [70]

Apart from the AP-2 adaptor complex, clathrin-coated vesicles also contain other proteins. A vast array of monomeric adaptors that bind to clathrin and are able to interact with integral membrane proteins, PtdIns( 4,5)P2 and AP-2, in order to ensure the occurrence of clathrin-mediated endocytosis of transmembrane proteins have been identified (*Fig. 6*). Examples of such adaptors include epsins, the CALM/AP180 protein, HIP1, and HIP1R. The N-terminal domain of these proteins that binds to PtdIns(4,5)P2 is called ENT H (epsin N-terminal homology) or ANT H (AP180 N-terminal homology domain). Another group of monomeric proteins includes Dab2, ARH, and Numb. These proteins bind to AP-2, and some of them interact with clathrin. They all contain a phosphotyrosine-binding domain (PTB) responsible for interaction with membrane lipids and for recognition of the FXNPXY motif localized in the cytoplasmic portions of LDLRs. It was demonstrated that phosphorylation of tyrosine at this motif is not a prerequisite for the binding of monomeric adaptors [71].

In addition to binding to cell membrane lipids, the adaptors recognize the signals localized in the cytoplasmic portion of the receptors. These may include posttranslational modifications (phosphorylation, ubiquitination), short peptide motifs, or both [86]. It is presumed that binding of the adaptor to the cytoplasmic membrane is stabilized upon simultaneous interaction of the adaptor with PtdIns(4,5)P2 and the receptor [70, 87].

The role of all these adaptor proteins is poorly understood. Perhaps, the use of various proteins enables to avoid simultaneous accumulation of various GPCR in the same clathrin-coated vesicle [88].

Specific recognition of the receptor for its subsequent removal from the membrane is the role of adaptors that has been most researched. This recognition leads to disruption of the delivery of the receptor to the ligand responsible for its activation and often to the transfer of the receptor to lysosomes for degradation. Internalization of the receptor mediated by a specific adaptor may also result in the receptor being directed to another cellular compartment with its own set of signaling molecules [89]. Another consequence of the selective removal of the receptor from the cell surface may be such a cellular development, which is associated with one of the daughter cells receiving a different set of adaptors responsible for the endocytosis of certain receptors. For instance, during the development of Drosophila sense organs, the adaptor Numb is transferred to only one of the two daughter cells. Numb is responsible for the adjustment of the development of the cell it is located in by inhibiting transduction of signals along the Notch-pathway. Inhibition is attributed to receptor endocytosis; however, it is unclear whether the protein Numb controls the endocytosis of Notch directly or via the transmembrane regulator of Notch – Sandopo [90, 91].

As mentioned above, the proteins Numb, Dab2, and ARH contain a domain that specifically recognizes the FXNPXY sequence in the cytoplasmic portion of the receptors. It was demonstrated that the proteins ARH and Dab2 are involved in the endocytosis of the LDL receptor, and the Numb protein regulates the endocytosis of the integral membrane proteins, including the EGF and Notch receptors [88].

The Epsin protein is involved in clathrin-mediated endocytosis in mammalian cells, where it plays an important role in the cytoplasmic membrane bending attributed to the action of the ENT H domain. The occurrence of this process is associated with the C-terminal portion of the protein binding to clathrin coat components (clathrin and AP-2 N-terminal domains, and EHdomain of the protein Eps15), which leads to the assembly of the clathrin lattice. The UIM-repeats of the epsin protein that acts as an adaptor protein facilitate recognition of the ubiquitinated “cargo”: in particular transmembrane proteins [88, 92].

The proteins AP180 and CALM ease the assembly of the clathrin-coated vesicles and regulate their size [20, 93, 94]. It is assumed that AP180 and CALM play an important role in ensuring the polarity and control of the growth of the axons and dendrites of the hippocampal neurons [95].

**TRIP8b – novel clathrin-binding protein and a potential endocytosis adaptor**

**Fig. 7:**
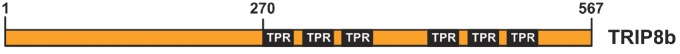
Schematic representation of the protein TRIP8b domain organization

IP8b (TPR-containing Rab8b interacting protein) is one of the recently discovered potential adaptors involved in the regulation of endocytosis. Being expressed predominantly in brain tissue, TR IP8b was initially identified as a protein that interacts with the small GTP-ase Rab8b [96]. Six TPR-motifs are located in the C-terminal part of TR IP8b, forming the TPR-domain (*Fig. 7*). TPR-motifs represent repeats consisting of 34 amino acids. These motifs are found in many proteins Fig 7. Schematic representation of the protein TRIP8b domain organization and are involved in protein-protein interactions [97]. These repeats are often arranged consecutively, resulting in a spatial structure consisting of two antiparallel α-helices connected by a short loop [98]. The N-terminal portion of TR IP8b does not contain sequences homologous to other known proteins and undergoes alternative splicing [99].

It is known that TR IP8b is 40% identical to the Pex5 protein (peroxin protein) and their C-terminal portion containing TPR-domains are 57% identical (other names for the TR IP8b include Pex5Rp, Pex5p related protein) [100]. The Pex5 protein is found in many organisms ranging from yeast to mammals; it is responsible for the recognition and import of peroxisomal proteins containing the C-terminal SKL motif (peroxisometargeting signal type 1, PTS1) from the cytosol into peroxisomes. Despite the fact that TR IP8b recognizes the PTS1 sequence it has been established that it is not involved in peroxisomal protein import [100].

TR IP8b interacts with the Rab8b protein and also directly with a protein that forms the HCN -channel (Hyperpolarization- activated, Cyclic Nucleotide-regulated channel) [99, 101]. It was found that TR IP8b binds to the CIRL1 receptor [102], which belongs to the GPCR class, and to the transmembrane protein called Caspr [103].

HCN channels belong to the family of voltage-dependent channels [104-106]. These channels are involved in the control of the heart and brain pacemakers’ activity, ensuring resting membrane potential and synaptic transmission (see reviews [107] and [108]). Investigations into the interactions between TR IP8b and the HCN channel demonstrated that TR IP8b regulates the functions and surface expression of the channel [99, 101, 109, 110]. It is assumed that TR IP8b acts as an auxiliary adaptor for HCN and that the interaction is mediated by at least two different regions in the TR IP8b and HCN molecules [111, 112].

All of the previously identified interactions of TR IP8b with other proteins were mediated by the TPR-domains localized in the C-terminal part of TR IP8b [96, 99, 100] or a segment located in the conserved central region of the protein [109, 111, 112].

As mentioned above, the N-terminal portion of TR IP8b undergoes alternative splicing. Isoforms of the protein generated as a result of splicing affect the HCN transport and its localization on the cytoplasmic membrane differently: some isoforms increase the surface expression of HCN 1 and others decrease it [109, 110]. It was found that TR IP8b interacts with clathrin and this interaction involves the N-terminal portion of the protein TR IP8b [103, 113]. The clathrin-binding site in the TR IP8b molecule consists of two short motifs reminiscent of the “clathrin box” motif.

In order to investigate the TR IP8b functions two types of knockout mice were obtained. The phenotype of mice lacking certain TR IP8b isoforms was identical to that of wild-type mice [114]. Abnormalities in the motor learning and increased resistance during the behavioral despair test were observed in mice with a complete absence of the protein (TR IP8b^-/-^) [115].

The need to conduct further investigations into clathrin-mediated endocytosis is unquestionable. Internalization of ligand-activated receptors and the functions of various accessory proteins are of significant interest. Interactions between clathrin and numerous adaptor proteins are currently being actively investigated. Clathrin is involved in various processes: endocytosis, intracellular traffic, and segregation of chromosomes. It is assumed that abnormalities in the functioning of clathrin can lead to the development of certain diseases. In this regard, investigations into the structure and functions of clathrin-coated vesicles, as well as the proteins involved in the formation of the latter, is of interest from the point of view of molecular biology and biomedicine.

## Abbreviations

EEA1: Early Endosome Antigen 1
GPCR: G-Protein-Coupled Receptor
GRK: G-proteincoupled Receptor Kinase
LDLR: Low-Density Lipoprotein Receptor
PtdIns(4,5)P2: phosphatidylinositol-4,5-bisphosphate
